# Effects of Resveratrol on Vitrified Porcine Oocytes

**DOI:** 10.1155/2013/920257

**Published:** 2013-10-07

**Authors:** Elisa Giaretta, Marcella Spinaci, Diego Bucci, Carlo Tamanini, Giovanna Galeati

**Affiliations:** Dipartimento di Scienze Mediche Veterinarie (DIMEVET), Università di Bologna, Via Tolara di Sopra 50, 40064 Ozzano dell'Emilia, Bologna, Italy

## Abstract

Vitrified MII porcine oocytes are characterized by reduced developmental competence, associated with the activation of the apoptotic pathway. Resveratrol (R), a polyphenolic compound present in several vegetal sources, has been reported to exert, among all its other biological effects, an antiapoptotic one. The aim of this study was to determine the effects of R (2 **µ**M) on the apoptotic status of porcine oocytes vitrified by Cryotop method, evaluating phosphatidylserine (PS) exteriorization and caspases activation. R was added during IVM (A); 2 h postwarming incubation (B); vitrification/warming and 2 h postwarming incubation (C); all previous phases (D). Data on PS exteriorization showed, in each treated group, a significantly higher (*P* < 0.05) percentage of live nonapoptotic oocytes as compared with CTR; moreover, the percentage of live apoptotic oocytes was significantly (*P* < 0.05) lower in all R-treated groups relative to CTR. The results on caspase activation showed a tendency to an increase of viable oocytes with inactive caspases in B, C, and D, while a significant (*P* < 0.05) increase in A compared to CTR was recorded. These data demonstrate that R supplementation in various phases of IVM and vitrification/warming procedure can modulate the apoptotic process, improving the resistance of porcine oocytes to cryopreservation-induced damage.

## 1. Introduction 

Vitrification of oocytes is the most recent cryopreservation methodology, used in different species such as human [1], bovine [2], goat, and swine [3].

Currently, several studies have been carried out to improve the efficacy of cryopreservation protocols, validating different cryoprotectant solutions, incubation times, oocytes containers, and other many conditions [4].

Recent progresses in the vitrification technique are attested by the high number of born after cryopreservation of human [5], mouse [6], cat [7], and bovine [8] oocytes, but no piglets have been obtained from cryopreserved swine oocytes so far.

Compared with other domestic species, the high intracellular lipid content [9] and the wide cell volume make porcine oocytes more susceptible to storage at low temperature, with a consequent decrease of oocytes survival rate and apoptotic progression after thawing [10, 11].

Moreover, the survival and development of unfertilized vitrified porcine oocytes are significantly lower than those of fertilized vitrified ones [12, 13]. During the vitrification/warming process many oocyte ultrastructures, such as mitochondria, smooth endoplasmatic reticulum, meiotic spindle, and plasma membrane, show considerable damages that contribute to reduce the developmental potential of oocytes after fertilization [14, 15].

In addition, oocytes that survive cryopreservation significantly reduce their glutathione (GSH) content and accumulate reactive oxygen species (ROS) [16].

ROS, such as superoxide anions (O^−^_2_), hydroxyl radicals (OH^−^), and H_2_O_2_, are generated during intermediate steps of oxygen reduction; their heap, also associated with the glutathione efflux, is one of the main factors which are effective in inducing the apoptotic activation, characterized by biochemical events that result in specific morphological changes including cell shrinkage and progressive DNA and cell membrane damage, ultimately leading to cell death. Signals to death receptors (extrinsic apoptotic pathway) or to mitochondria (intrinsic apoptotic pathway) concur in the activation of caspases, a family of cysteine proteases with similar aminoacid sequences, structure, and specificity that promote morphological and biochemical cell changes, typical of apoptosis [17, 18]. Finally, phosphatidylserine (PS), that is normally confined to the inner plasma membrane leaflet, after apoptotic stimuli is externalized and subsequently recognized by specific PS receptors of macrophages and other phagocytic cells, inducing apoptotic cells engulfment [19–21].

Therefore, one of the current challenges to reproductive cryobiologists is to prevent oocytes degeneration in order to maintain their developmental competences.

To preserve the antioxidant defence system in oocyte, specific substances that play antioxidant roles, such as ascorbate [22], epigallocatechin-3-gallate [23], *β*-mercaptoethanol [13], anthocyanin [24], and trans-*ε*-viniferin [25], were added during in vitro oocytes/embryo culture and storage.

Recently, treatment of porcine oocytes with 2 *μ*M Resveratrol during IVM [26] reduces the intracellular level of ROS and increase GSH concentration in matured oocytes, resulting in increase of blastocyst development after parthenogenetic activation (PA) and in vitro fertilization (IVF).

Resveratrol, through its simultaneous activity on multiple molecular targets, is effective in modulating different cell pathways and, depending on its concentration, and its effect may be reversed [27]. Resveratrol has been reported to act as antioxidant because of its ability to decrease mitochondria ROS production, scavenge superoxide radicals, inhibit lipid peroxidation, and regulate the expression of antioxidant cofactors and enzymes [28].

On thise basis we decided to add 2 *μ*M Resveratrol in maturation, vitrification, and postvitrification media in order to determine its effect on apoptotic process and oocytes viability. 

For this purpose the externalization of phosphatidylserine using Annexin V (Annexin V/Hoechst 33342/PI) and the caspase activation through FITC-VAD-FMK staining (FITC-VAD-FMK/Hoechst 33342/PI) were evaluated.

## 2. Materials and Methods

Unless otherwise specified, all the reagents were purchased from Sigma-Aldrich (Milan, Italy). Cryotops (Kitazato, Fuji, Japan) were obtained from BioCare Europe (Roma, Italy).

### 2.1. In Vitro Maturation (IVM) of Cumulus-Oocyte Complexes (COCs)

Ovaries were collected at a local abattoir and transported to the lab within 2 h in a thermos filled with physiological saline at 30–35°C. COCs from follicles 3–6 mm in diameter were aspirated using a 18-gauge needle attached to a 10 mL disposable syringe. Under a stereomicroscope, intact COCs were selected and transferred into a petri dish (35 mm, Nunclon, Denmark) prefilled with 2 mL of modified PBS supplemented with 0.4% BSA. Only COCs with complete and dense cumulus oophorus were used. After three washes in NCSU 37 [29] supplemented with 5.0 mg/mL insulin, 0.57 mM cysteine, 10 ng/mL epidermal growth factor, 50 *μ*M *β*-mercaptoethanol, and 10% porcine follicular fluid (IVM medium), groups of 50 COCs were transferred to a 4-well multidish (Nunclon) containing 500 *μ*L of the same medium per well and cultured at 39°C in a humidified atmosphere of 5% CO_2_ and 7% O_2_ in N_2_. During the first 22 h of maturation, the IVM medium was supplemented with 1.0 mM db-cAMP, 10 IU/mL eCG (Folligon, Intervet, Boxmeer, The Netherlands), and 10 IU/mL hCG (Corulon, Intervet). After culturing for 22 h, COCs were transferred to fresh maturation medium and cultured for a further 24 h period [29].

### 2.2. Oocyte Vitrification with Cryotops and Warming

The protocol of vitrification with Cryotop carrier and solution has been described by Kuwayama et al. [30, 31]. Briefly, denuded oocytes (*N* = 5) were transferred into equilibration solution (ES) consisting of 7.5% ethylene glycol (EG) and 7.5% dimethylsulfoxide (DMSO) in Hepes-buffered Ham's F10 (HF10; Gibco, Invitrogen, Monza, Italy) and 20% fetal calf serum (FCS; Gibco) at 39°C for 5–15 min. Thereafter, oocytes were transferred into 20 *μ*L drops of vitrification solution (VS) consisting of 15% EG, 15% DMSO, and 0.5 M sucrose dissolved in HF10 and 20% FCS. After incubation for 20–30 s, oocytes were loaded on Cryotop and plunged into liquid nitrogen (LN2). The entire process, from exposure in VS to plunging into LN2, was completed within 45–60 s. Vitrified oocytes were warmed by submerging vitrification devices directly into 39°C thawing solution (1.0 M sucrose dissolved in HF10 and 20% FCS) for 1 min, and then they were transferred to a dilution solution (0.5 M sucrose dissolved in HF10 and 20% FCS) for 3 min. Subsequently, oocytes were washed twice for 5 min in washing solutions (HF10 supplemented with 20% FCS) before being transferred in IVM medium for 2 h.

### 2.3. Annexin V Staining of Phosphatidylserine Residues

The Annexin V binding assay was employed to detect phosphatidlserine (PS) externalization on the plasma membrane. Annexin V, a member of the phospholipid-binding annexin family, binds most efficiently to PS, which is externalized on the outer plasma membrane of cells exposed to apoptotic stimuli. 

Oocytes were washed three times with Dulbecco's phosphate-buffered saline (DPBS) and transferred to 100 *μ*L of binding buffer (Alexa Fluor 488 Annexin V/Dead Cell Apoptosis Kit, Molecular Probes, Eugene, USA) with 5 *μ*L of Alexa Fluor Annexin V, 1 *μ*L of propidium iodide (PI) (100 *μ*g/mL), and 0.2 *μ*L of 5 mg/mL Hoechst 33342 (Ho) for 20 min at 39°C in the dark. After incubation, the oocytes were washed three times in binding buffer and then mounted on glass slides which were examined with an Eclipse E 600 (Nikon Europe BV, Badhoevedorp, The Netherlands) epifluorescence microscope equipped with a digital camera.

**Figure 1:**
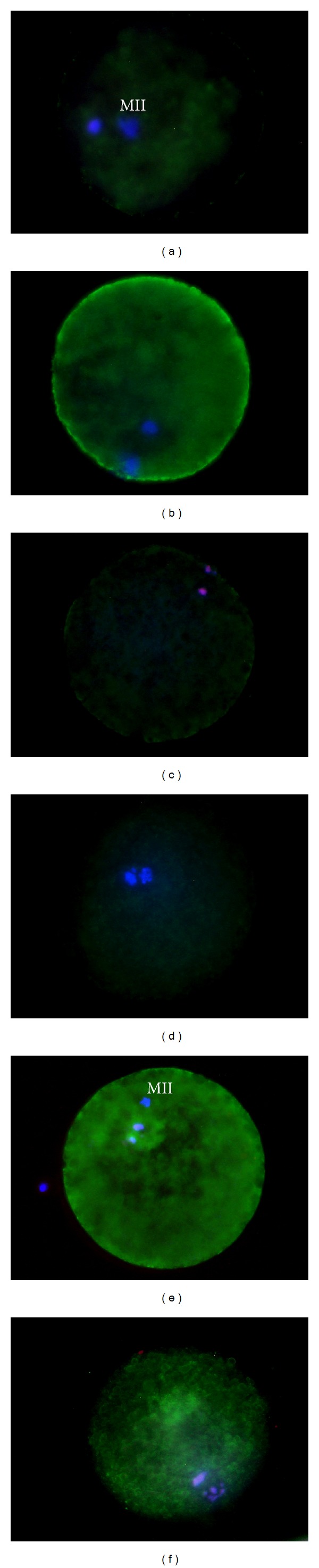
((a)–(c)) Fluorescent micrographs of oocytes after combined staining with Annexin V/Hoechst 33342 (Ho)/PI. (a) Live non apoptotic oocytes with Ho positive nuclei and no annexin staining (A−/PI−); (b) Live apoptotic oocytes with Ho positive nuclei and annexin positive signal on the membrane (A+/PI−); (c) Dead oocyte which showed PI positive red nuclei (PI+). ((d)–(f)) Representative fluorescent micrographs of pig oocytes after FITC-VAD-FMK, Ho and PI staining. (d) Live non apoptotic oocytes with Ho positive nuclei and no FITC-VAD-FMK staining (VAD−/PI−); (e) Live apoptotic oocyte with the cytoplasm stained in green by FITC-VAD-FMK (VAD+/PI−); (f) Dead oocyte with the metaphase plate stained in red by PI (PI+).

Oocytes were classified as follows:

- live nonapoptotic oocytes with Ho-positive nuclei and no annexin staining (A−/PI−) (Figure 1(a)),
- live apoptotic oocytes with Ho-positive nuclei and annexin-positive signal on the membrane (A+/PI−) (Figure 1(b)),
- necrotic oocytes which showed PI-positive red nuclei, indicative of membrane damage with or without annexin staining on the membrane (PI+) (Figure 1(c)).

### 2.4. Assessment of Activated Caspases

The activation of caspases was detected through FITC-VAD-FMK (Molecular Probes, Leiden, The Netherlands); VAD-FMK is a cell permeable caspase inhibitor that covalently binds activated caspases, conjugated to FITC. Oocytes were washed twice with DPBS and then incubated with 500 *μ*L of DPBS containing 1 *μ*M of FITC-VAD-FMK and 0.2 *μ*L of 5 mg/mL Ho for 30 min at 39°C. During the final 5 min of VAD-FMK/oocyte incubation, 3 *μ*L of a solution 1 mg/mL of propidium iodide were added to detect dead oocytes. Oocytes were then washed twice for 5 min with DPBS and mounted on glass slides. Samples were assessed by fluorescence microscopy. Stained oocytes were classified in three groups:

- viable oocytes without active caspases (VAD−/PI−)(Figure 1(d)),
- viable oocytes with FITC-VAD-FMK positivity, indicative of caspase activation (VAD+/PI−) (Figure 1(e)),
- dead oocytes (PI+) (Figure 1(f)).

### 2.5. Experimental Design

All oocytes were submitted to IVM, vitrification, and warming procedures and were evaluated after incubation for 2 h into maturation medium.

Oocytes were divided into the following 5 experimental groups:

 (CRT) without Resveratrol addition;
 (A) 2 *μ*M Resveratrol supplementation during IVM;
  (B) 2 *μ*M Resveratrol supplementation during the postwarming incubation for 2 h;
 (C) 2 *μ*M Resveratrol supplementation during vitrification/warming and 2 h post-warming;
 (D) 2 *μ*M Resveratrol supplementation in all previous steps.

### 2.6. Statistical Analysis

Each experiment was repeated at least 3 times. All statistical analyses were performed using R version 2.15.2. [32]. Chi square test was performed and the level of significance was set at *P* < 0.05.

## 3. Results 

### 3.1. Experiment 1: Detection of Apoptosis by Annexin V Labeling

The addition of R, in all treated groups, induced a percentage of live nonapoptotic oocytes (A−PI−) significantly higher (*P* < 0.05) than nontreated control (Table 1). Moreover, in all groups supplemented with R the percentage of live apoptotic oocytes (A+PI−) was significantly (*P* < 0.05) lower than in CTR group. Finally, R supplementation in B, C, and D groups significantly (*P* < 0.05) reduced the percentage of dead oocytes (PI+) compared to CTR.

### 3.2. Experiment 2: Assessment of Activated Caspases

The results on caspases activation showed a tendency to an increase of viable oocytes with inactive caspases (VAD−PI−) in B, C, and D groups, while a significant (*P* < 0.05) increase in A group compared to CTR was recorded.

No significant variations were observed in the percentage of live oocytes with active caspases (VAD+/PI−), while a significant reduction of dead oocytes (PI+) was detected in A group compared to CTR (Table 2). 

## 4. Discussion

While ascorbate [22] and *β*-mercaptoethanol [13] have been already assayed in vitrification-warming solutions, in mouse embryos and porcine oocytes, respectively, this study, for the first time, tested Resveratrol in the improvement of oocyte cryopreservation. Ascorbate (0.1 mmol/l) has been demonstrated to reduce the levels of hydrogen peroxide in mouse embryos, increasing the inner cell mass when added in slow-freezing or vitrification solutions; *β*-mercaptoethanol (50 *μ*mol/l) decreased ROS activity but did not improve viability and fertilization ability of vitrified-warmed MII oocyte, while significantly increased blastocyst formation ability of porcine oocytes vitrified after in vitro fertilization.

Our results demonstrate that 2 *μ*M R in IVM and vitrification-warming phases increases oocytes viability, modulating the apoptotic process. Annexin V labeling showed a significant increase in live nonapoptotic oocytes and a parallel reduction of live apoptotic oocytes in all groups added with R as compared with CTR groups. 

FITC-VAD-FMK staining evidenced a tendency to an increase of viable oocytes with inactive caspases in all R-treated groups compared with CTR; a significant difference was recorded only between A and CTR groups. However, the percentage of viable oocytes with active caspases was not affected by R treatment. 

Several recent studies demonstrated that PS-mediated phagocytosis can occur without caspases activation [33–35]. Thus, we can hypothesize that the different trends in viability and apoptosis observed in annexin V and FITC-VAD-FMK assays may depend on an involvement of caspases and PS externalization in the whole apoptotic process, with a prevalent action of R in one of these two events.

Our results show that R mainly influences PS exteriorization, rather than caspases activation. This R effect can be related to GSH increase, observed by Kwak et al. [26] after the addition of the same R concentration to IVM solution. This hypothesis is supported also by He et al. [36], who demonstrated that inhibition of GSH efflux had no effect on the activation of caspases 3, 8, and 9, but decreased the translocation of PS. Cellular GSH homeostasis plays a crucial role in radical scavenging activity [18] and in cytoplasmatic maturation of porcine oocyte [37–39]. Somfai et al. [16] reported that low intracellular GSH levels and high H_2_O_2_ concentration in vitrified porcine oocytes, besides other ultrastructural cryodamages, increase the sensitivity to oxidative stress at the beginning of embryo culture, reducing porcine oocytes development and subsequent male pronucleus formation.

In addition, several studies demonstrated the ability of R to scavenge ROS [40, 41] and to modulate intracellular GSH depletion or synthesis, in relation to its concentration [42–44]

Therefore we can suppose that R could be able to maintain GSH homeostasis, with a subsequent inhibition of PS externalization. 

Our results on R effect on caspase activation, which is not as evident as PS externalization in vitrified oocytes assayed after 2 h incubation after thawing, agree well with those by Vallorani et al. [11], who observed that caspase activation seems to be a reversible phenomenon. That work showed a significant reduction of vitrified live oocytes presenting a faint FITC-VAD-FMK staining after 2 h of postwarming incubation, compared to those observed immediately after warming; moreover, no significant differences were detected, in the same time lapse, in the number of apoptotic oocytes (A+P−) as assayed by Annexin V staining. Taken together, our and those results seem to suggest that 2 h of post-warming incubation may be beneficial in modulating the caspase cascade that can be further reduced by R addition, in a significant manner when added in IVM solutions.

A previous study [45] demonstrated that in neuronal cells the externalization of PS occurs during the late phase of apoptosis while caspase activation begins in the early one. 

Therefore, we can hypothesize that while caspase activation can be arrested after 2 h post warming incubation, PS externalization may be a tardive and irreversible apoptotic event that could be avoided by R addition in one or more steps of the IVM and vitrification procedure.

In our study we did not observe any algebraic sum of positive effects of Resveratrol when added during vitrification/warming and 2 h of culture after warming (C group) or in the whole IVM-vitrified-warming-postwarming process (D group). Resveratrol could be immediately oxidized, modulating the GSH redox balance and thus increasing the oocyte-reducing power. Therefore, it seems that it might be employed either in IVM, in vitrified-warming, or 2 h post warming solutions, in order to prevent, inhibit, or repair cryoinjury damages.

## 5. Conclusions

In conclusion, the present results confirm the occurrence of vitrification-induced oocyte injuries, as previously reported, and suggest to improve vitrification protocols by Resveratrol addition.

Supplementation with 2 *μ*M Resveratrol in IVM, vitrification-warming, or 2 h postwarming solutions could improve and optimize the quality and the resistance of IVM porcine oocytes to cryopreservation, modulating cell apoptotic process.

Other polyphenolic compounds could help in minimizing cryodamage and in optimizing current Cryotop vitrification method, improving the success of applications in female gamete preservation.

## Figures and Tables

**Table 1:** Effect of Resveratrol supplementation on exteriorization of phosphatidylserine, assayed by Annexin V/Hoechst 33342/PI staining, in vitrified oocytes. Data are presented as mean percentage.

Group	*N* oocytes	% viable oocytes (A− PI−)	% apoptotic oocytes (A+ PI−)	% dead oocytes (PI+)
CTR	310	66.1^a^	28.4^a^	5.5^a^
A	150	78.7^b^	15.3^b^	6^a^
B	124	83.1^b^	16.9^b^	0^b^
C	122	80.3^b^	18.9^b^	0.8^b^
D	114	81.6^b^	17.6^b^	0.9^b^

Different superscripts within the same column indicate significant differences (*P* < 0.05).

CTR: control group; (A) 2 *μ*M R supplementation during IVM; (B) 2 *μ*M R supplementation during the postwarming incubation for 2 h; (C) 2 *μ*M R supplementation during vitrification/warming and 2 h after warming; (D) 2 *μ*M supplementation in all previous steps.

**Table 2:** Effect of Resveratrol on caspase activation in vitrified oocytes, as assayed by FITC-VAD-FMK/Hoechst 33342/PI staining. Data are presented as mean percentage.

Group	*N* oocytes	% live oocytes (PI−)		% dead (PI+)
		VAD−	VAD+	
CTR	165	81,82^a^	10,30^a^	7,88^a^
A	84	91,67^b^	7,14^a^	1,19^b^
B	76	86,84^ab^	9,21^a^	3,95^ab^
C	75	84,00^ab^	13,33^a^	2,67^ab^
D	91	89,01^ab^	7,69^a^	3,30^ab^

Different superscripts within the same column indicate significant differences (*P* < 0.05).

CTR: control group; (A) 2 *μ*M R supplementation during IVM; (B) 2 *μ*M R supplementation during the postwarming incubation for 2 h; (C) 2 *μ*M R supplementation during vitrification/warming and 2 h after warming; (D) 2 *μ*M supplementation in all previous steps.
